# Sarcoidosis-like Skin Lesions as the First Manifestation of Ataxia-Telangiectasia

**DOI:** 10.3390/children12060672

**Published:** 2025-05-23

**Authors:** Borko Milanovic, Gordana Vijatov-Djuric, Andrea Djuretic, Jelena Kesic, Vesna Stojanovic, Milica Jaric, Ognjen Ležakov

**Affiliations:** 1 Medical Faculty of Novi Sad, University of Novi Sad, Hajduk Veljkova 3, 21000 Novi Sad, Serbia; borko.milanovic@mf.uns.ac.rs (B.M.); gordana.vijatov-djuric@mf.uns.ac.rs (G.V.-D.); jelena.kesic@mf.uns.ac.rs (J.K.); vesna.stojanovic@mf.uns.ac.rs (V.S.); 2 Institute for Child and Youth Healthcare of Vojvodina, Hajduk Veljkova 10, 21000 Novi Sad, Serbia; andrea.djuretic@izzzdiovns.rs (A.D.); 1351d19@mf.uns.ac.rs (M.J.)

**Keywords:** chronic granulomatous skin disease, ataxia-telangiectasia, immunodeficiency, cerebellar degeneration

## Abstract

Ataxia-telangiectasia is a rare autosomal recessive disorder that is difficult to diagnose due to its unpredictable presentation. It is characterized by cerebellar degeneration, telangiectasias, immunodeficiency, frequent pulmonary infections, and tumors. Immune system abnormalities manifest as disruptions in both cellular and humoral immunity. The most common findings include decreased levels of immunoglobulin classes (IgA, IgM, IgG, and IgG subclasses) and a reduced number of T and B lymphocytes. A four-year-old girl was initially evaluated and treated for skin lesions that presented as crusts spreading across her body. She was monitored by a pulmonologist due to frequent bronchial obstructions. Over time, she developed bilateral scleral telangiectasia, saccadic eye movements, and impaired convergence. Her gait was wide-based and unstable, with truncal ataxia and a positive Romberg sign. Laboratory tests revealed decreased immunoglobulin G levels, subclass IgG4 levels, elevated alpha-fetoprotein, and a reduced number of T and B lymphocytes. Brain magnetic resonance imaging showed cerebellar atrophy. Whole-exome sequencing identified heterozygous variants c.1564-165del, p.(Glu5221lefsTer43), and c.7630-2A>C in the serine/threonine-protein kinase ATM (ataxia-telangiectasia mutated) gene, confirming the diagnosis of ataxia-telangiectasia. Following diagnosis, treatment with intravenous immunoglobulin replacement was initiated along with infection prevention and management. The goal of this case report is to raise awareness of the atypical initial presentation that may lead to a diagnostic delay. We emphasize the importance of considering ataxia-telangiectasia in the differential diagnosis, even when classical neurological signs are not yet evident.

## 1. Introduction

Ataxia-telangiectasia (A-T) is a rare autosomal recessive disorder caused by mutations in the ATM gene, which encodes a serine/threonine-protein kinase [1]. The incidence of this disease ranges between 1:40,000 and 1:300,000, affecting both sexes equally [2]. A-T is challenging to diagnose due to its unpredictable presentation. It is characterized by cerebellar degeneration, telangiectasias, immunodeficiency, frequent pulmonary infections, and tumors [3]. Immune system abnormalities include cellular and humoral immune dysfunction. The most common findings are reduced levels of one or more immunoglobulin classes (IgA, IgM, IgG, and IgG subclasses) and decreased numbers of T and B lymphocytes [4,5].

Definitive diagnosis of A-T is made through genetic testing to identify ATM gene mutations. However, even in rare cases where genetic testing is unavailable, a clinical, laboratory, and radiological evaluation can suggest the disease [3].

There is currently no specific therapy for A-T. Management includes intravenous immunoglobulin replacement therapy, infection prevention, and treatment, which help reduce complications and extend life expectancy [6].

## 2. Case Description

**Figure 1:**
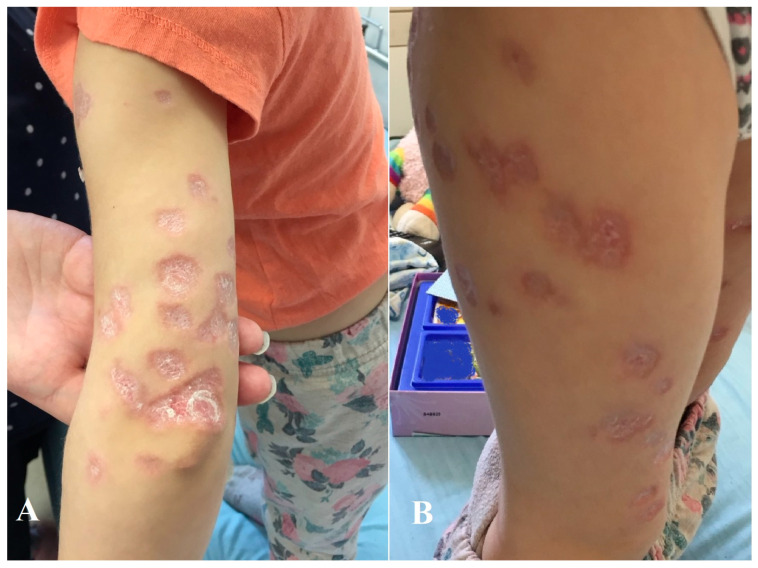
Cutaneous granulomatous lesions on the lateral and posterior part of the upper left arm (**A**) and on the anterior part of the right thigh (**B**) clinically mimicking sarcoid-like dermatoses. The lesions presented as erythematous to livid firm nodules with central desquamation. Images were taken on the same day during the same stage of the disease.

We present the case of a nine-year-old girl who had been monitored by dermatologists since the age of two years due to livid skin lesions with desquamation (Figure 1).

Over time, the lesions expanded and deepened. At four years old, she was hospitalized in the Institute for Child and Youth Health Care of Vojvodina due to worsening skin lesions. Her medical history included frequent pneumonia and bronchial obstructions, requiring follow-ups by a pulmonologist.

**Figure 2:**
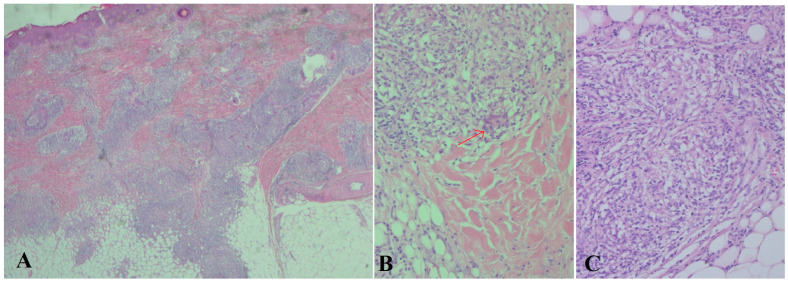
Chronic granulomatous dermatitis and lobular panniculitis. (**A**) Microphotography of skin biopsy with granulomatous inflammation in dermis and in subcutaneous adipose tissue (HE × 50). (**B**) Detail of granuloma with giant cell (HE × 200). the arrow represents a granuloma with giant cell. (**C**) Granulomatous panniculitis (HE × 200).

She was admitted to the hospital for diagnostic evaluation of her general condition and assessment of cutaneous lesions. During hospitalization, laboratory findings revealed elevated angiotensin-converting enzyme (ACE) levels (2097 nkat/L; reference range < 1867). A chest computerized tomography (CT) scan showed subpleural micronodules in the right lung and fibrotic bands in the left, while the rest of the findings were within normal limits. Brain magnetic resonance imaging (MRI) was performed and showed no significant pathological changes. Since the skin lesions worsened, a biopsy was performed. A biopsy of the skin and of the muscle from the left thigh was obtained, fixed in formalin and embedded in paraffine blocks in total, and stained by Periodic acid–Schiff (PAS), hematoxylin and eosin (HE), and Giemsa and Gommory methods. The biopsy of the muscle was within normal limits, with uniform fiber size, preserved striations, and peripherally nuclear position. In the interstitial tissue, a small amount of mature adipose cells were observed, with normal capillary blood vessels. The skin had preserved epidermis with discrete acanthosis and well-defined epidermal rete ridges. In the deep reticular dermis and in the upper part of the subcutaneous adipose tissue, mainly in lobular parts, coalesced granulomas were observed. The granulomas consisted of epithelioid cells, a few giant cells of foreign body type and of Langhans type, and a small number of lymphocytes and plasma cells. In the biopsy material, there was no necrosis or vasculitis within the granulomas. The diagnosis corresponds to chronic granulomatous dermatitis and panniculitis (Figure 2).

Based on these findings, including histopathology, sarcoidosis was suspected, and oral prednisone and methotrexate therapy was initiated, leading to the regression and healing of the skin lesions. Residual scaring was present at the sites of the former lesions.

During a subsequent hospitalization for bronchial obstruction, laboratory tests revealed decreased IgG levels (4.09 g/L (ref. range 5–13), prompting the initiation of immunoglobulin replacement therapy.

At the age of five years, additional clinical findings emerged, including bilateral scleral telangiectasia, saccadic eye movements, impaired convergence, a wide-based unstable gait, truncal ataxia, and a positive Romberg sign. These findings led to another hospitalization for further diagnostics. The cutaneous lesions showed no further progression.

The findings highlighted elevated levels of alpha-fetoprotein, −197.1 IU/mL (ref. range < 7.3) as well as decreased values of the IgG4 subclass, <0.05 g/L (ref. range 0.01–1.699). Immunophenotyping of T and B lymphocytes revealed that the absolute number of T lymphocytes was reduced relative to the reference range for age, while the absolute number of B lymphocytes was extremely low.

**Figure 3:**
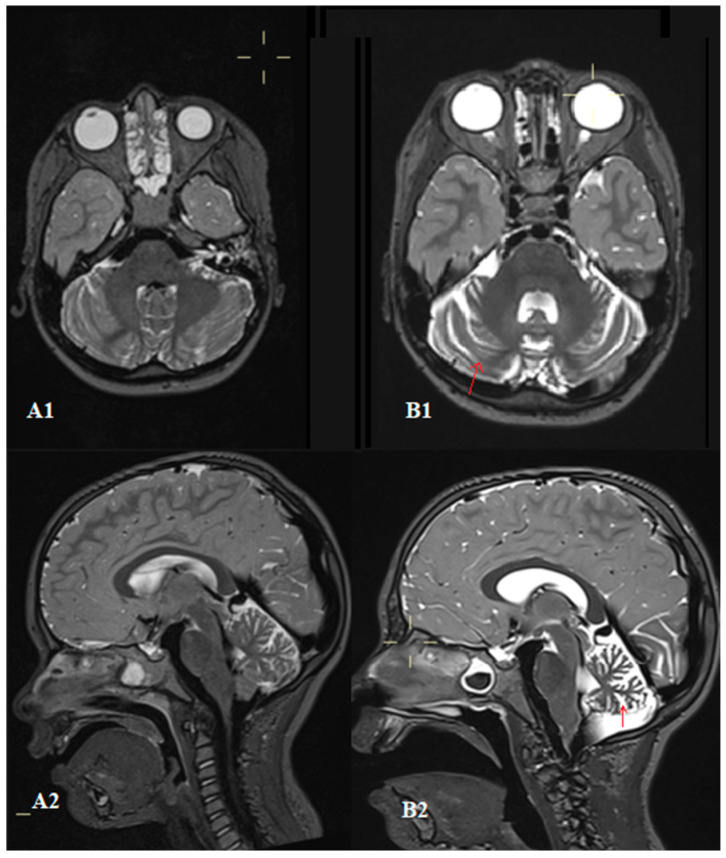
T2-weighted MRI sequences of the brain demonstrate bilateral cerebellar hemispheric and vermis atrophy (**B1**,**B2**) in comparison to prior imaging performed three years before, when these structures appeared normal (**A1**,**A2**). The red arrow represents cerebellar athrophy.

Due to neurological deterioration, several MRIs of the brain were performed, revealing, for the first time, pronounced volume reduction in both cerebellar hemispheres and the vermis at the age of seven years, three years after the initial MRI and two years after the onset of neurological symptoms. Perivascular gliosis of the white matter was also noted (Figure 3).

Upon reviewing all results and considering the clinical presentation, a suspicion of ataxia-telangiectasia (A-T) was raised. Genetic testing was conducted, identifying heterozygous variants c.1564-165del, p.(Glu5221lefsTer43), and c.7630-2A>C in the serine/threonine-protein kinase ATM gene.

Following comprehensive laboratory and genetic investigations and brain MRI, a diagnosis of ataxia-telangiectasia was established. Corticosteroid and methotrexate therapy was discontinued, and intravenous immunoglobulin replacement therapy continued along with infection prevention and treatment. The cutaneous lesions remained unchanged.

## 3. Discussion

A-T, also known as Louis–Bar syndrome, affects multiple organ systems, leading to progressive cerebellar ataxia, oculocutaneous telangiectasia, recurrent pulmonary infections, immunodeficiency, and radiation sensitivity. Clinical manifestations vary significantly in onset and progression, complicating the diagnosis [7]. In pediatric patients, A-T may present as either a typical early onset or an atypical late-onset form [3]. Typically, ataxia is noticeable when a child begins sitting and standing, which was not the case in our patient since the ataxia was first noticed at the age of five years [8].

In addition to the aforementioned symptoms, a variety of cutaneous manifestations can be observed in A-T, such as mucocutaneous telangiectasias, vitiligo, and café-au-lait spots and also premature graying of the hair [9]. Chronic granulomatous skin disease is also described as a manifestation of the condition, and according to literature data, only 10% of individuals with A-T will develop chronic granulomatous disease. This highlights the diverse clinical presentation of A-T and the challenges in raising suspicion for this rare disorder [10]. In healthy individuals, granulomas typically arise as a response to infection or the presence of foreign bodies; however, the exact mechanism underlying granuloma formation in A-T remains unclear. One theory suggests that a deficiency of B lymphocytes and naive T cells leads to increased activity of NK cells, resulting in immune dysregulation of macrophages, which play a key role in the initiation and maintenance of granulomatous disease. According to another theory, granulomatous skin disease is associated with increased activity of γδ T cells, which are the main source of interferon-gamma and tumor necrosis factor [11]. In this case, chronic granulomatous skin disease was an early sign. The patient’s skin lesions showed mild regression after corticosteroid therapy, as reported in previous studies [10].

Telangiectasias usually appear between ages five and eight years, with bulbar conjunctiva being the most common site, though they may later affect other body regions [12]. In our patient, ocular findings included bulbar telangiectasia, saccadic eye movements, and impaired convergence, consistent with A-T [13].

Laboratory findings supportive of A-T include elevated alpha-fetoprotein, reduced immunoglobulin classes, and decreased T and B lymphocyte counts [14]. The majority of patients exhibit both cellular and humoral immune deficiencies. Immunodeficiency is typically diagnosed within the first five years of life and does not tend to progress over time [15]. According to the literature data, decreased levels of IgG subclass 2 are associated with a poorer prognosis and faster disease progression [16]. In our patient, reduced levels of IgG subclass 4 were observed.

Immune dysregulation leads to pulmonary complications, which become increasingly common during the second decade of life and represent the leading cause of mortality in individuals with A-T. The most frequent pulmonary manifestations include acute and chronic respiratory infections, bronchiectasis, bronchitis, and chronic lung disease, all of which result in impaired pulmonary function, as was observed in our patient [17].

MR imaging plays a crucial role in diagnosing A-T, since it is the method of choice, especially in the pediatric population, due to the absence of radiation exposure and its superior visualization of the brain and spinal cord. Initial neuroimaging studies may appear unremarkable; however, as the disease progresses, MRI of the brain typically reveals progressive and diffuse cerebellar atrophy [18]. In our case, the initial brain MRI was normal, while follow-up imaging showed marked volume loss of both cerebellar hemispheres as well as the vermis, consistent with A-T.

A-T patients are at risk for malignancies, primarily leukemias and lymphomas in childhood and solid tumors in adulthood [1]. No malignancies were detected in our patient.

The definitive diagnosis of A-T is established by genetic testing, specifically the identification of homozygous or compound heterozygous mutations in the ATM gene [19]. In our case, two heterozygous variants were identified: c.1564-165del, p.(Glu5221lefsTer43), and c.7630-2A>C in the serine/threonine-protein kinase gene.

There is currently no curative treatment for this disease; therefore, the therapeutic approach is primarily symptomatic. Early identification and close monitoring by a multidisciplinary team—including immunologists, pulmonologists, neurologists, and physiatrists—are essential to delay the onset of complications for as long as possible [20].

## 4. Conclusions

The diverse initial presentation of A-T can obscure an early diagnosis. This case highlights the diagnostic challenges posed by atypical initial presentations of ataxia-telangiectasia, particularly when cutaneous manifestations preceded neurological symptoms for several years. A comprehensive diagnostic approach proved essential. Clinicians should consider A-T in patients presenting with any characteristic features of the disease, such as the recurrent sinopulmonary infections, progressive ataxia, and oculocutaneous telangiectasias. A combination of said clinical symptoms, laboratory findings (elevated alpha-fetoprotein and reduced immunoglobulin levels), and radiological findings (cerebellar atrophy) are key to differentiating A-T from other conditions. Genetic testing is crucial for diagnosis, as the definitive diagnosis was achieved through next-generation sequencing, which revealed a homozygous pathogenic variant in the ATM gene. Early recognition of such presentations can facilitate timely genetic counseling, supportive care, and targeted monitoring strategies to improve outcomes in affected individuals.

## Data Availability

Data is contained within the article.

## Abbreviations

The following abbreviations are used in this manuscript:

ACE	angiotensin-converting enzyme
A-T	ataxia-telangiectasia
ATM	ataxia-telangiectasia mutated
CT	computerized tomography
H&E	hematoxylin and eosin
Ig	immunoglobulin
MRI	magnetic resonance imaging
PAS	Periodic acid–Schiff
